# Hemophilic pseudotumor of the knee joint: Emphasizing prevention and early diagnosis in a rare disease

**DOI:** 10.1002/ccr3.8822

**Published:** 2024-04-30

**Authors:** Shritik Devkota, Sugat Adhikari, Samiksha Lamichhane, Dipendra Adhikari, Bhola Bika, Shayeri Roy Choudhary, Tajinder Bhalla

**Affiliations:** ^1^ Department of Radiodiagnosis and Imaging Anil Baghi Hospital Punjab India; ^2^ Shreegaun Primary Health Care Center Dang Nepal; ^3^ Department of Radiodiagnosis and Imaging B. P. Koirala Institute of Health Sciences Dharan Nepal; ^4^ Tuberculosis Treatment Center Pokhara Nepal; ^5^ Tokha Chandeshwari Hospital Tokha Saraswati Nepal; ^6^ Department of Radiology Aster DM Healthcare Dubai UAE; ^7^ Department of Orthopedics Anil Baghi Hospital Punjab India

**Keywords:** hemophilia, hemophilic pseudotumor, knee joint, multimodality imaging, lytic bone lesion

## Abstract

**Key Clinical Message:**

Hemophilic pseudotumors are rare complications occurring in individuals with severe hemophilia, characterized by progressive cystic swellings in muscles and/or bones due to recurrent bleeding. Timely initiation of factor VIII replacement is crucial.

**Abstract:**

Hemophilic pseudotumors are rare complications occurring in individuals with severe hemophilia, characterized by progressive cystic swellings in muscles and/or bones due to recurrent bleeding. Although their incidence has decreased with the advent of factor VIII replacement therapy, they still create challenges, particularly in regions with limited access to medical care. Here, we present a case report of a hemophilic pseudotumor of the knee joint in a 15‐year‐old male with hemophilia A. The patient presented with severe left knee pain, swelling, and restricted range of motion, prompting further investigation. Imaging studies revealed lytic lesions, and MRI bone signal changes consistent with hemophilic pseudotumors. Prompt initiation of factor VIII replacement therapy and supportive management led to a significant improvement in symptoms and joint functionality. Follow‐up after 2 months showed that the swelling had significantly reduced in size, with marked improvement in the functionality of the knee joint. This case confirms what is already known in the hemophilia literature: how important it is to prevent, diagnose, and treat pseudotumors early in hemophilia. However, longer clinical and imaging follow‐up of this case is necessary to determine whether the complaints associated with pseudotumors resolve with hematologic treatment or will require surgical treatment.

## 1 INTRODUCTION

Hemophilia A and B, both X‐linked clotting disorders, result from a deficiency in factors VIII and IX, respectively. The clinical presentation varies based on disease severity. Severe hemophilia is characterized by spontaneous bleeding into joints and muscles, which occurs usually when the level of Factor VIII is below 1% of its normal value.^1^, ^2^ Over time, repeated joint bleeds lead to painful and debilitating hemophilic arthropathy. The elbows, knees, and ankles are the most commonly affected joints.^3^ Pseudotumors are rare complications in hemophiliacs, involving chronic encapsulated blood collections due to recurrent bleeding in bones or soft tissues. As these swell, pressure within the hematoma damages nearby structures.^4^ Hemophilic pseudotumors have significantly decreased in both frequency and severity due to the utilization of factor VIII replacement therapy.^5^, ^6^ Nevertheless, this complication can persist in developing nations, where access to medical services remains limited. We report a case of hemophilic pseudotumor in the bones of the knee joint in a 15‐year‐old male.

## 2 CASE REPORT

### 2.1 Case history and examination

**FIGURE 1:**
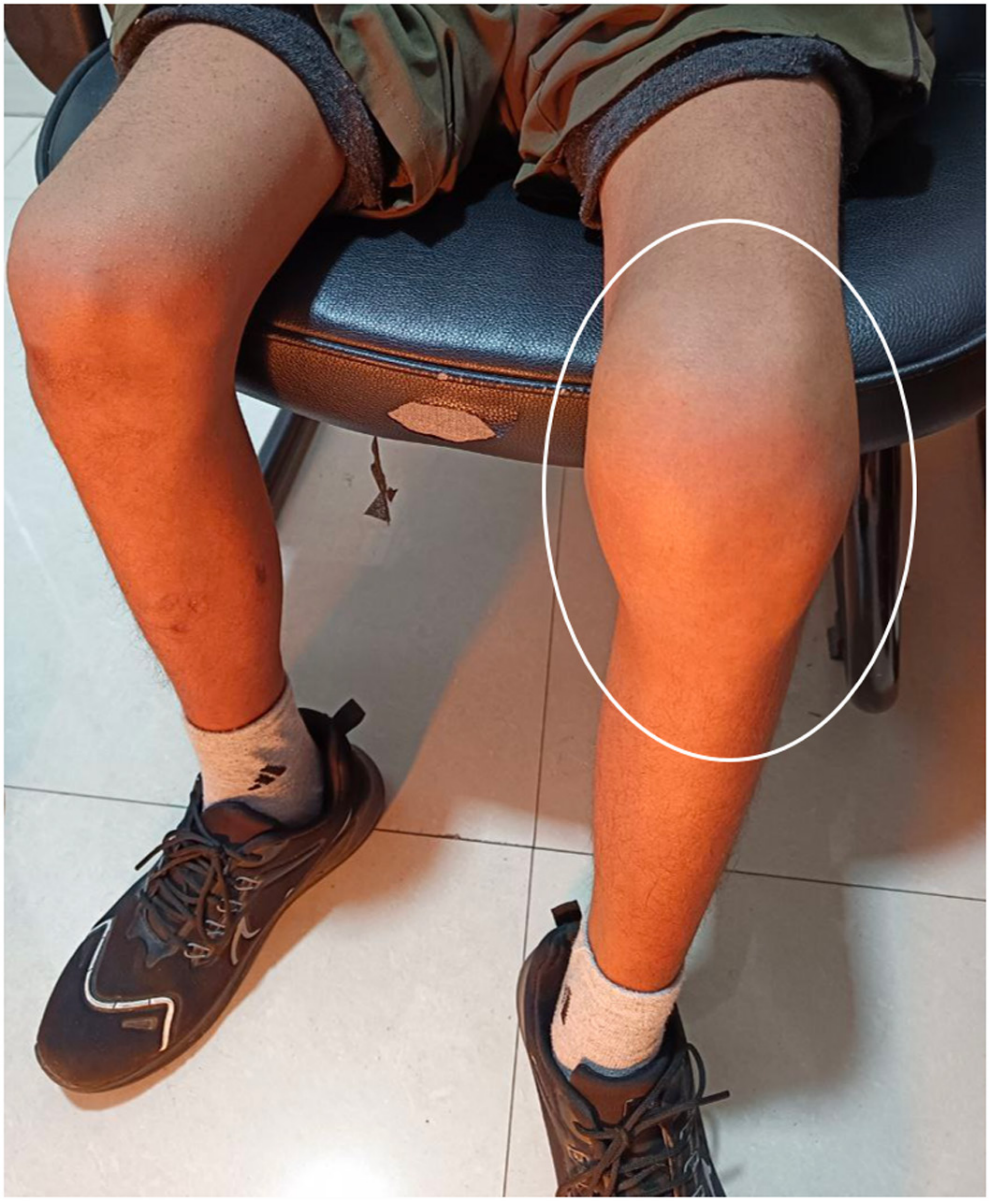
Clinical image of the patient showing diffuse swelling of the left knee joint (white circle) and normal right knee joint.

A 15‐year‐old male with a known history of hemophilia A presented to the orthopedic clinic complaining of severe left knee pain, swelling, and restricted range of motion over the past 3 weeks. Patient has reported that the pain and swelling began a week ago following a fall from a bicycle, and his symptoms were progressively worsening, particularly exacerbated with weight‐bearing activities. Clinical examination revealed diffuse swelling, warmth, and tenderness around the left knee joint, with limited range of motion noted, especially in flexion and extension. Clinical picture of the left knee joint is highlighted in Figure 1. Neurovascular examination was carried out to rule out any grave complication, due to the significant swelling, but was normal. Blood tests showed a normal complete blood count but revealed an elevated activated partial thromboplastin time (aPTT), consistent with his underlying hemophilia A.

### 2.2 Methods

**FIGURE 2:**
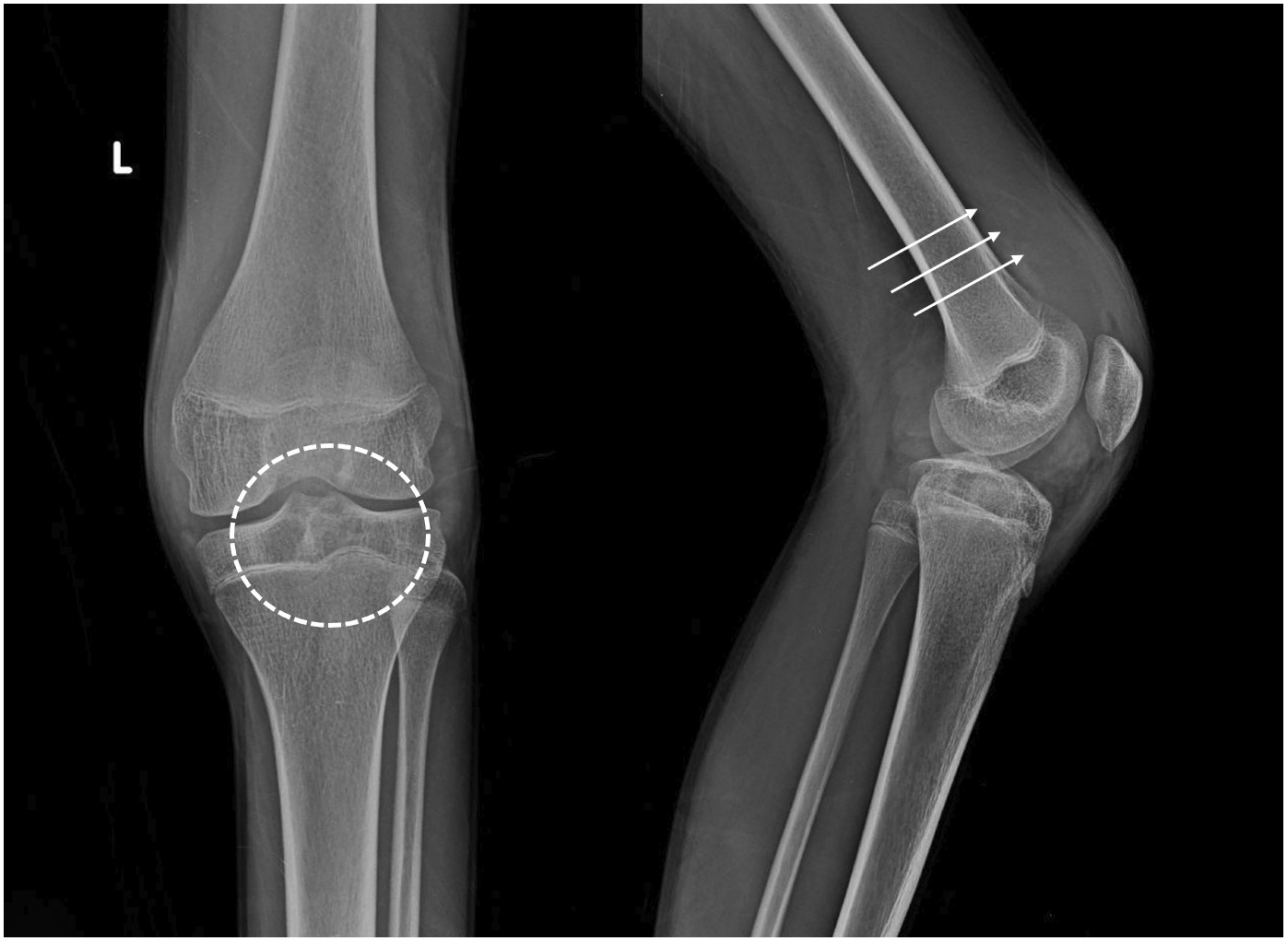
Knee radiograph showing lytic area in intercondylar eminence and along lateral tibial plateau (dotted black circle). Distension in supapatellar region with fat pad separation suggesting suprapatellar bursal fluid (white arrows).

**FIGURE 3:**
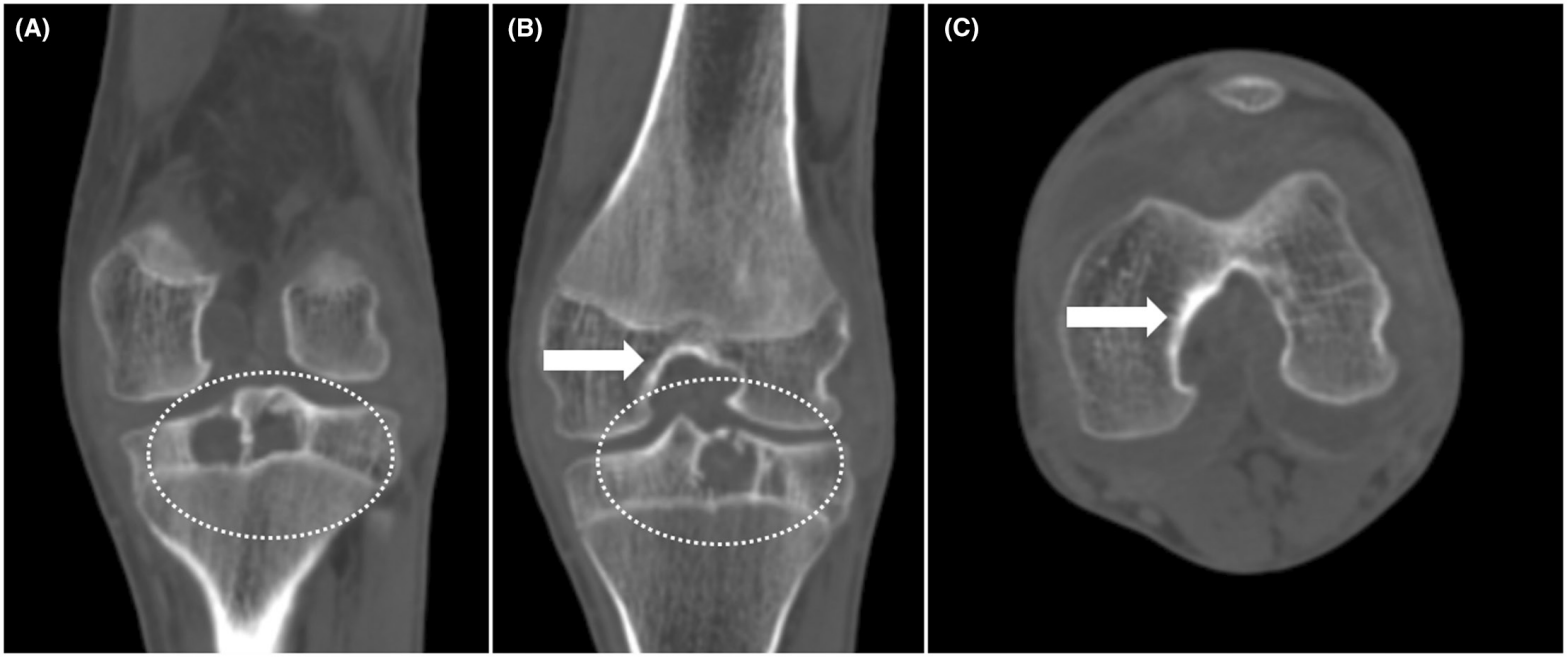
Coronal reconstructed (A, B) and axial (C) non contrast CT images of knee joint showing lytic lesions in tibial plateau (dotted circles) and widened intercondylar notch (block arrows).

**FIGURE 4:**
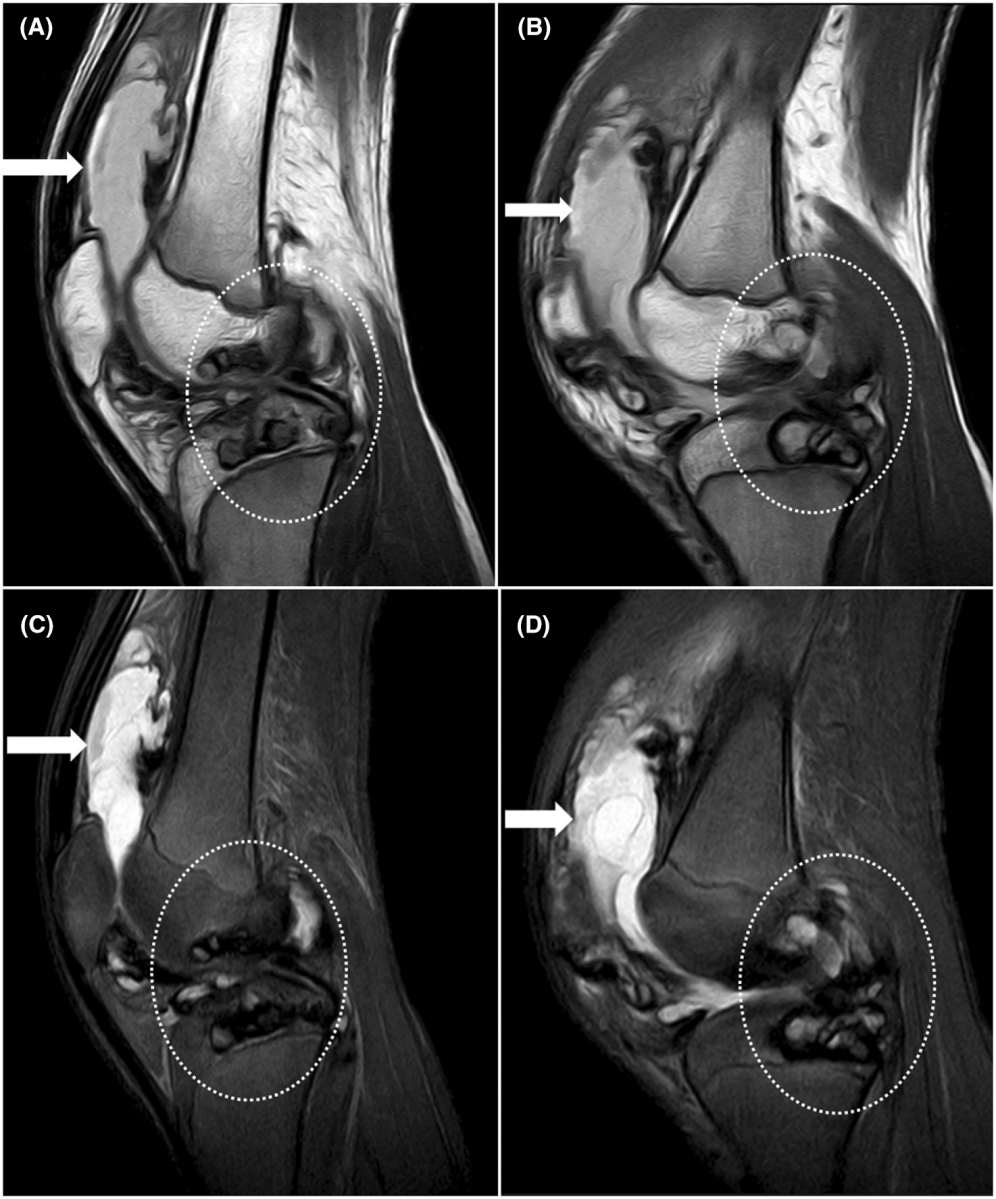
T1W (A, B) and PD (C, D) sagittal MRI images showing suprapatellar T1/PD hyperintense effusion (block white arrows) in patellofemoral compartment extending to suprapatellar recess suggesting hemorrhagic effusion. T1 and PD heterogeneously hyperintense lesions (dotted white arrows) in tibial plateau and medial femoral epicondyle suggesting hemorrhagic contents‐hemophilic pseudotumor.

**FIGURE 5:**
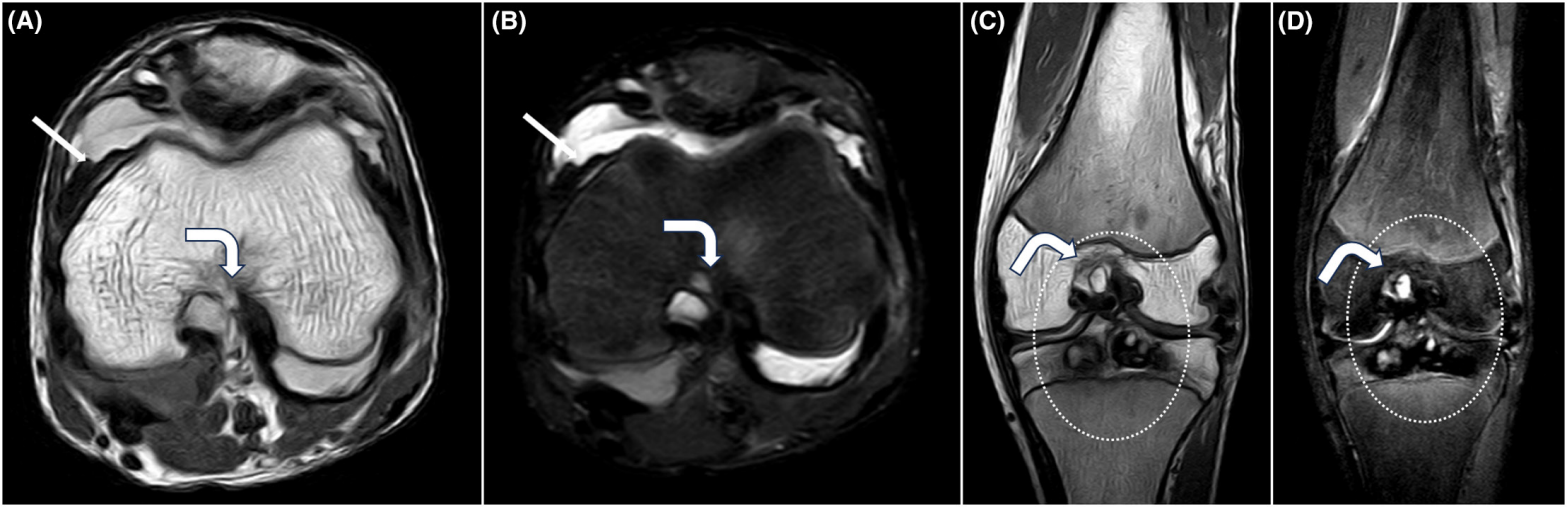
Axial [T1W] (A), [PD] (B) and Coronal [T1W] (C), [PD] (D) images showing widened intercondylar notch (bent white arrows) with heterogeneously hyperintense lesions in tibial plateau (dotted white circle) and hypointense synovial thickening (straight white arrows).

Plain radiograph of the left knee joint was done and it showed a lytic area in intercondylar eminence and along lateral tibial plateau and distension in suprapatellar region with fat pad separation suggesting suprapatellar bursal fluid. (Figure 2). For further characterization and plan of management, non‐contrast CT (Figure 3) and MRI (Figure 4, Figure 5) were advised given the nature of the history and physical examination leading to the radiological diagnosis of hemophilic pseudotumors involving the bones of the knee joint. While non‐contrast CT showed lytic lesions in tibial plateau, MRI revealed hemorrhagic joint effusion with T1 and PD heterogeneously hyperintense lesions in tibial plateau and medial femoral epicondyle suggesting hemophilic pseudotumors with hemorrhagic contents.

### 2.3 Conclusion and results

Treatment was promptly initiated, including factor VIII replacement therapy to control bleeding and inflammation, along with pain management using analgesics and NSAIDs. Knee immobilization was suggested for the first week of the treatment to facilitate decrease in the swelling and physical therapy was commenced to improve joint function and strength. Regular follow‐up appointments were scheduled to monitor the pseudotumor size and assess treatment response. Over the following weeks, our patient responded well to the treatment regimen, with a gradual decrease in pain and swelling noted. Follow‐up after 2 months showed that the swelling had significantly reduced in size, with marked improvement in the functionality of the knee joint. His lab values, along with PT, aPTT, and factor VIII assay were also within normal limits.

This case confirms what is already known in the hemophilia literature: how important it is to prevent, diagnose, and treat pseudotumors early in hemophilia. However, longer clinical and imaging follow‐up of this case is necessary to determine whether the complaints associated with pseudotumors resolve with hematologic treatment or will require surgical treatment.

## 3 DISCUSSION

Hemophilic pseudotumors are rare complications occurring in less than 2% of patients with severe hemophilia, characterized by progressive cystic swellings in muscle and/or bone due to repeated bleeding.^7^ In 1918, Starker provided the initial description of pseudotumors.^8^ Multiple studies have investigated hemophilic pseudotumors, examining their diverse origins, clinical presentations, and management modalities.^9^ However, the evidence of hemophilic pseudotumor occuring in the bones of the knee joint are relatively rare in the literature.

Hemophilic pseudotumors manifest initially as painless, solid masses that adhere to deeper tissues. Though initially asymptomatic, they can later lead to pathological fractures. Typically, these pseudotumors appear as slowly expanding, enclosed cystic masses, and some patients recall a prior injury before their emergence. These masses often restrict mobility due to their size and location. Furthermore, the skin overlaying them may undergo necrosis, leading to infection, or the masses may rupture, resulting in swift bleeding.^10^, ^11^, ^12^ It can result in joint deformities, necrosis of soft tissues, and compartment syndromes. Volkmann's contractures frequently arise in individuals with hemophilia due to extensive hemorrhage within forearm muscles. Moreover, ankle contractures might develop, requiring prompt intervention. Furthermore, these pseudotumors can lead to bone deterioration, formation of fistulas, and damage to peripheral nerves.^9^, ^13^, ^14^

Hemophilic pseudotumors are diagnosed and characterized through a combination of methods including physical examination, X‐rays, sonography, CT scans, MRI, and blood tests. Among these, MRI is deemed highly effective for detecting and diagnosing HP, aiding in treatment decisions, and evaluating treatment outcomes. It allows monitoring of blood product evolution, lesion size in hard‐to‐reach areas, and recurrent bleeding in chronic lesions. A special type of MRI sequence, called gradient echo T2‐weighted, is commonly used for hemophilic joint evaluation. It can detect hemosiderin and cartilage changes. Using a contrast agent like gadolinium isn't consistently helpful for hemophilic arthropathy. Different MRI scales, like Denver and European, were developed to track joint damage and compare treatments. These scales rate joint changes, such as fluid and cartilage loss, to monitor progression. Both scales show good agreement among different readers and for repeated readings.^7^, ^12^, ^13^, ^15^, ^16^

Several treatment protocols are available, each presenting their own advantages and drawbacks. Combining replacement therapy with immobilization proves beneficial for small, recently developed pseudotumors. Ultrasound‐guided aspiration is appropriate for pseudotumors containing fluid content. While curettage can decrease the pseudotumor mass, it carries risks such as persistent fistula, recurrence, or infection. Radiotherapy targets unresectable lesions by impacting blood vessels and endothelial proliferation. Intra‐arterial embolization helps in reducing bleeding during surgery. Surgical intervention, particularly for proximal pseudotumors, stands as the most effective approach. Nevertheless, due to the distinctive nature of this condition and the limited number of clinical cases, managing complex hemophilic pseudotumors remains a challenge.^9^, ^10^, ^16^

## 4 CONCLUSION

While hemophilic pseudotumors have become less prevalent in regions where factor VIII replacement therapy is readily accessible, they persist as a significant challenge in areas with limited healthcare resources. Timely initiation of factor VIII replacement therapy is crucial not only for controlling bleeding and inflammation but also for preventing complications such as joint deformities and soft tissue necrosis. This should be done along with adequate anti‐inflammatory medications, physiotherapy and possibly surgical management. Ensuring equitable access to factor VIII replacement therapy in developing nations can substantially improve the quality of life and long‐term outcomes for individuals with hemophilia, highlighting the urgent need for global efforts to address healthcare disparities and ensure access to essential treatments for all individuals, regardless of geographical location, or socioeconomic status.

## AUTHOR CONTRIBUTIONS

**Shritik Devkota:** Conceptualization; data curation; writing – original draft; writing – review and editing. **Sugat Adhikari:** Conceptualization; data curation; writing – original draft; writing – review and editing. **Samiksha Lamichhane:** Conceptualization; data curation; writing – review and editing. **Dipendra Adhikari:** Writing – original draft; writing – review and editing. **Bhola Bika:** Writing – original draft. **Shayeri Roy Choudhary:** Conceptualization; data curation; writing – original draft. **Tajinder Bhalla:** Conceptualization; data curation; supervision; writing – original draft.

## FUNDING INFORMATION

The authors declare that they have no known competing financial interests or personal relationships that could have appeared to influence the work reported in this paper.

## CONFLICT OF INTEREST STATEMENT

The authors have declared that no competing interests exist.

## ETHICS STATEMENT

The authors declare that the procedures were followed according to the regulations established by Clinical Research and Ethics Committee and to the Helsinki Declaration of the World Medical Association updated in 2013.

## CONSENT

Written informed consent was obtained from the patient to publish this report in accordance with the journal's patient consent policy.

## Data Availability

The datasets analyzed during the current study are available from the corresponding author upon reasonable request. Additionally, comprehensive literature sources used for the literature review are cited appropriately within the manuscript.
